# Context Matching is not Reasoning: Assessing Generalized Evaluation of Generative Language Models in Clinical Settings

**DOI:** 10.21203/rs.3.rs-7325383/v1

**Published:** 2025-08-29

**Authors:** Andrew Wen, Qiuhao Lu, Yu-Neng Chuang, Guanchu Wang, Jiayi Yuan, Jiamu Zhang, Liwei Wang, Sunyang Fu, Kurt D. Miller, Heling Jia, Steven D. Bedrick, William R Hersh, Kirk E. Roberts, Xia Hu, Hongfang Liu

**Affiliations:** The University of Texas Health Science Center at Houston; The University of Texas Health Science Center at Houston; Rice University; Rice University; Rice University; Rice University; The University of Texas Health Science Center at Houston; The University of Texas Health Science Center at Houston; Mayo Clinic; Mayo Clinic; Oregon Health & Science University; Oregon Health & Science University; The University of Texas Health Science Center at Houston; Rice University; The University of Texas Health Science Center at Houston

## Abstract

Current discussion surrounding the clinical capabilities of generative language models (GLMs) predominantly center around multiple-choice question-answer (MCQA) benchmarks derived from clinical licensing examinations. While accepted for human examinees, characteristics unique to GLMs bring into question the validity of such benchmarks. Here, we validate four benchmarks using eight GLMs, ablating for parameter size and reasoning capabilities, validating via prompt permutation three key assumptions that underpin the generalizability of MCQA-based assessments: that knowledge is applied, not memorized, that semantic consistency will lead to consistent answers, and that situations with no answers can be recognized. While large models are more resilient to our perturbations compared to small models, we globally invalidate these assumptions, with implications for reasoning models. Additionally, despite retaining the knowledge, small models are prone to answer memorization. All models exhibit significant failure in null-answer scenarios. We then suggest several adaptations for more robust benchmark designs more reflective of real-world conditions.

## Introduction

Recent attention surrounding large language models (LLMs) has driven great interest in utilizing such models for clinical purposes^1,2^, particularly in the context of learning healthcare systems. A prerequisite to practical adoption, is, however, evaluation: an open question when discussing LLMs that covers a multitude of dimensions and considerations^3^. Historically, the evaluation of artificial intelligence and machine learning (AIML) models for clinical tasks was done task-specifically due to implementation considerations and technical limitations of pre-LLM models. Specifically, such models generally required finetuning for adaptation for differing tasks^4–6^ and were cheap to train or fine-tune while requiring costly annotated datasets^7,8^. The incentives surrounding evaluating such models can thus be characterized as datasets being expensive, model inference being cheap, and model training being moderately costly, with model training to fit the dataset always being required.

LLMs represent a drastic shift, being computationally intensive and costly to run, requiring substantial hardware and time investment, and expensive to fine-tune. Concurrently, their popularity lies in generalizability across a wide range of tasks without the need for extensive fine-tuning. This shift is further driven by growing interest in small language models^9–11^ (SLMs) and multi-agent systems^12–15^, driven in large part by the high, vertically-scaling, resource requirements of large, widely generalizable, language models. Given the wide variety^16^ of models that exist today, each with their own specializations, and the fact that each model still represents a significant resource and time investment, it is helpful to have a general characterization of their clinical performance to subset viable candidates for further evaluation. Consequent to these incentives, there has been a shift towards using general-purpose benchmarks that aim to assess a model’s overall clinical competency and reasoning ability prior to engaging in task-specific fine-tuning and evaluation.

Among these general evaluation strategies, multiple-choice question answer (MCQA) benchmarks have emerged as the predominant methodology. Benchmarks such as the US Medical Licensing Examination (USMLE)^17^ and datasets like MedQA^18^ and MMLU^19^ are frequently employed to assess model performance due to their wide availability, straightforward evaluation obviating engagement of clinical domain expertise, and simple implementation. Additionally, these benchmarks align with human clinical licensure processes, further lending credence to claims on model clinical capability. As a result, much of the discussion surrounding LLMs centers on their competitive performance on these MCQA benchmarks, which has led to scrutiny regarding their validity and applicability^20^.

Beyond concerns^21^ on memorization and applicability to generation tasks, licensing examinations inherently assess general reasoning rather than disease-specific expertise, assuming that possession of requisite knowledge enables clinicians to arrive at accurate diagnoses and treatments through reasoning. Models, however, differ fundamentally from humans. Human strength lies in problem-solving, not in flawless knowledge retention, and we trust that if the requisite clinical knowledge is present, the human can consistently apply a reasoning process to arrive at the correct answer. Conversely, models excel at context matching but have shown limitations in reasoning fidelity^22^, particularly with reasoning models leveraging integrated chain-of-thought-based approaches^23^. Fundamentally, this difference therefore challenges the construct validity of MCQAs as an assessment method.

Nevertheless, MCQAs are still valuable: the straightforward evaluation and wide coverage remains one of the best ways to quickly capture a general sense of model clinical capabilities without significant time/effort investment, particularly as interest shifts to amalgamations of smaller specialized models. In this study, we therefore aim to assess the validity of the assumptions underpinning generalizability of MCQA-style benchmarks across both model size and reasoning capabilities, highlight their limitations when used to infer real-world clinical competency, and determine the degree to which contextual cues provided by the MCQA format impact the generalizability of the model’s observed performance.

## Results

**Figure 1:**
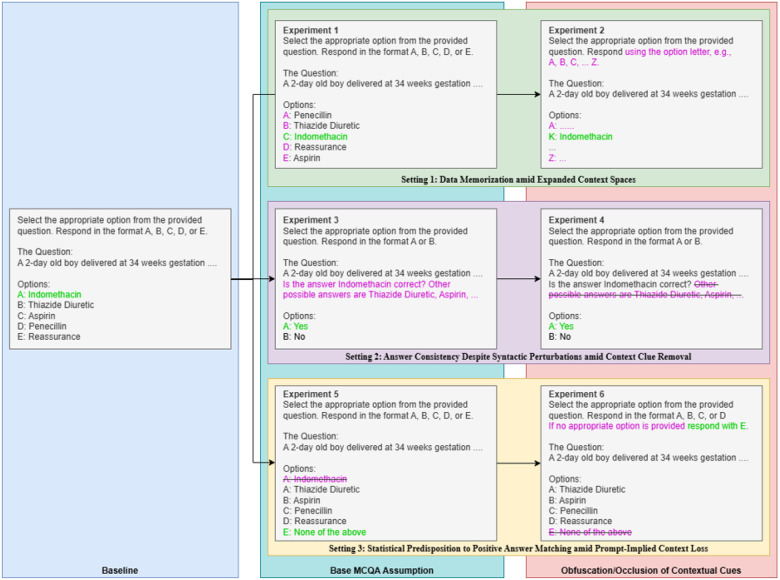
Experimental Settings and Generation Prompts

We test three baseline assumptions that underpin MCQA generalizability:1) that a successful answer implies that requisite knowledge is **possessed, retrieved**, and, in particular, **applied** to the problem, rather than just being memorized, 2) that the same semantic problem will lead to the same answer despite syntactic permutation, and 3) given the same knowledge, the examinee can identify when there is no correct answer presented in the options. Additionally, we assess the models’ dependence on the restricted search space afforded by the multiple-choice format by obfuscating or occluding context for each of the three assumptions. These assumptions are tested via prompt permutations (Fig. 1) on benchmark questions that models were able to answer correctly in original form.

**Figure 2:**
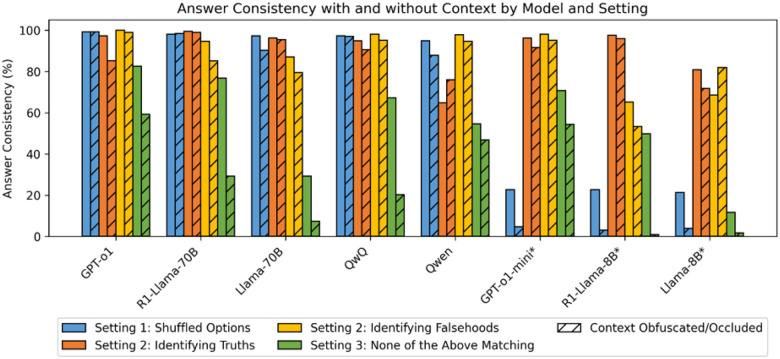
Answer Consistency with and without Context by Model and Setting

We show the baseline accuracy for our datasets of the various models assessed in this study is in Table 1, and the answer consistency upon permutation of the respective MCQA assumptions (color), alongside the impact of context availability (hash) for each of our varying experimental settings in Fig. 2.

While strong consistency is maintained for most large models (except for Qwen-2.5–32B) in Setting 2 when identifying truths with full context, substantial drops are observed for GPT-o1 and QwQ, and slight drops for other models. Interestingly, Qwen-2.5–32B consistency increases, despite this increase not being reflected in its reasoning model variant (QwQ). We note that the pattern is the opposite for identifying falsehoods: while GPT-o1 and QwQ remain relatively consistent with and without context, R1-Llama-70B and Llama-70B both experience substantial inconsistency. This trend is reflected for SLMs, although we note an increase in consistency without the occluded context for LLaMA-8B.

All models show substantive inconsistency for setting 3 even if a “None of the Above” option is explicitly provided, and < 40% consistency for most models if the “None of the Above” option is instead supplied as a prompt instruction.

**Figure 3:**
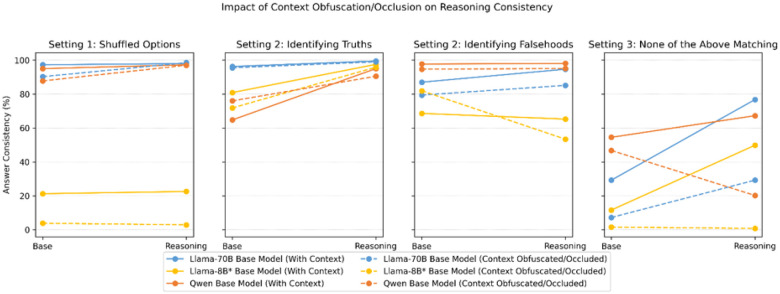
Impact of Context Obfuscation/Occlusion on Answer Consistency with and without Reasoning

In Fig. 3, we show the impact of context availability on answer consistency as a function of reasoning models vs. their respective base models.

While we generally observe an improvement in consistency (irrespective of whether context is provided) with the addition of reasoning capabilities, this is not globally true. Specifically, we note that adding reasoning does not affect consistency for small models in Setting 1, and the reasoning process hurts consistency in Settings 2 and 3 when context is removed.

**Figure 4:**
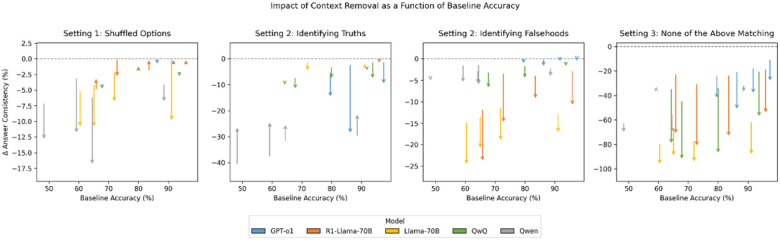
Impact of Context Obfuscation/Occlusion on Answer Consistency as a Function of Baseline Accuracy

Finally, in Fig. 4 we show the impact of these individual experimental settings and context availability on the overall dataset performance as a function of the dataset’s difficulty to the individual model (i.e., their baseline performance on the model). Due to figure scale, small models are not included in this figure, although they follow the same trends. We observe a correlation between baseline performance and answer consistency for all experimental settings, with the magnitude of inconsistency upon context obfuscation/occlusion being model dependent but baseline accuracy independent.

## Discussion

MCQA is accepted as part of the clinical licensure process due to an expectation that, while it is impossible to comprehensively cover all clinical scenarios in any reasonable assessment, it is sufficient to test retained knowledge. This validity of this expectation is underpinned by the human ability to consistently apply knowledge in supporting reasoning. Our experimental settings aimed to systemically evaluate both the extent of retained knowledge, as well as the application of said retained knowledge to actual clinical problem solving.

All large generative language models meet the reported USMLE passing threshold of 60%^37,38^ for human examinees, although several small models do not. Such performance clearly demonstrates a competitive level of clinical knowledge retention on par with human examinees. Our various experiments, however, surface several concerns with respect to application of such knowledge to problem solving. Here, we will discuss these concerns, as well as potential solutions to better adapt MCQA-based benchmarks to the era of data driven models.

### On the Applicability of Assumptions Underlying MCQA as an Assessment Method for Generative Language Models

The context-matching inherent to MCQA problems inherently tests knowledge retention, as correct responses require the model to generate appropriate statistical priors. While large models remain largely consistent in Setting 1, small models do not, suggesting significant reliance on data memorization for successful solves. Unlike LLMs which can **possess**, **retrieve**, and **apply** the requisite knowledge, the SLMs skip the last step. The requisite knowledge is retained, but it is not applied: instead, the answer is directly parroted without adaptation to the shuffled option context. Consequently, this brings significant concerns to the generalizability of the evaluation performance on these benchmarks for SLMs. As small models are often distilled from large, this inconsistency/memorization also undermines these assessments on LLMs.

We generally observe SLM inconsistency for Setting 2, invalidating the underlying assumption that syntactic permutations of the same semantic content will lead to the same response. Interestingly, however, the consistency, despite dropping, remains much higher than in Setting 1 for small models. While it is quite evident that data memorization is occurring for successful solves in the traditional multiple-choice context, this finding suggests that the small models do largely **possess**, and can **retrieve** the requisite knowledge for successful solves, and variations in the prompt syntax are capable of “tricking” the language model to not leverage the memorized shortcut and instead **apply** the knowledge correctly.

Concerningly, all models, irrespective of big or small, but particularly pronounced in non-reasoning models, show substantial inconsistency in Setting 3, indicating models strongly prefer choosing an available option even when none apply. This fundamentally contradicts the assumption that the model is truly applying the knowledge possessed, and is a dangerous limitation in clinical contexts given the possibility for unexpected inputs in clinical decision support contexts.

These observed inconsistencies fundamentally challenge the validity of MCQAs as an assessment tool for language models, particularly when model parameter size is small. The blatant data leakage and reliance on answer memorization challenges the generalizability of the model’s capabilities. Moderate inconsistency in responses with syntactic but without semantic variation challenges the assumption that possessed knowledge is consistently applied, and the strong preference for a positive answer highlights a major weakness of current models in option-selection settings. In a clinical context, these limitations have a direct impact on the very reason we accept multiple-choice questions as an assessment tool for generalized clinical competency, leading to inflated perceptions of model performance.

### On the Impact of Contextual Cues amid Reasoning Models

The removal of contextual cues will near universally negatively impact answer consistency (Fig. 3). This is itself concerning as real-world problems can have, depending on use-case, vastly expanded contexts. For instance, expanded contexts (as is the case in setting 1’s context expansion experiment) is a realistic use case as real-world decision making is not limited to a 4-option set. Similarly, it is not always realistic to have present a background listing of “what other items to consider” (which in and themselves are often designed to contain hints to the correct answer) in the prompt itself in real world scenarios as is the case in setting 2’s context occlusion experiment.

With respect to the impact of such occlusion and/or obfuscation on reasoning models, when ablated between reasoning/non-reasoning models within the same model family, reasoning models occasionally display greater inconsistency than their non-reasoning counterparts, particularly for models with smaller parameter sizes (Fig. 2), i.e., the reasoning process does not always help improve, but can rather hurt, consistency when context is removed. While this can partially be attributed to higher baseline performance, we posit that the internal chain-of-thought process inherent to reasoning models acts as self-reinforcement by serving as a statistical prior, amplifying inconsistency with loss of context.

### On Improving Model Consistency and MCQA as an Assessment Method

Despite the limitations on their generalizability, MCQAs remain valuable for evaluation in the clinical field due to their unambiguous scoring, efficiency, and lower domain expertise requirements compared to generative methods. It would therefore be beneficial to identify strategies to mitigate the MCQA drawbacks identified in this study while still maintaining the MCQA format. We examine this problem through two lenses: improving the model itself to address these consistency issues, and improving MCQA as an assessment to better reflect real-world conditions.

With respect to the former, we note that consistency is roughly positively correlated with baseline performance. There is reasonable suspicion that some of these inconsistencies are caused by learned shortcuts causing regurgitation rather than a lack of knowledge (Setting 1 vs Setting 2). Given that our various settings are all permutations that can be autonomously generated via symbolic rulesets, it may be beneficial to introduce these symbolic permutations into the model training process itself within the respective training set to, in essence, unlearn the shortcuts and instead encourage the models to apply the knowledge correctly, improving model consistency and potentially generalizability.

In regards to mitigating the impact of context occlusion and obfuscation, it was observed (Fig. 4) that the magnitude of inconsistency increase across the various experimental settings upon context obfuscation/occlusion is independent of the baseline accuracy of the model on said benchmarks, suggesting that the magnitude of inconsistency and dependence on contextual cues is a function of training strategy rather than the data on which the model is trained on. It may be beneficial to further investigate the interplay between these two factors.

With respect to the latter, multiple-choice questions are written to be relatively unambiguous by design. Given the strong dependence on limited option space demonstrated by Setting 1 context removal, it may be beneficial to introduce red herring distractor options (that are close to, but are not, the correct answer). To break memorization, beyond option shuffling, synonymous representations for both individual options and clinical entities within the questions can be substituted via autonomous means (e.g., via named entity recognition/entity linking). Issues of syntactic permutation of the same semantic content (Setting 2) can be resolved by symbolically permuting the syntax of the question in various manners (much as we do here in Setting 2) and giving a weighted accuracy score instead based on the agreement/correctness across these multiple permuted prompts. False-positive/hallucination rate (Setting 3) should be explicitly measured via introduction of null-answer variants of the questions as we present here.

Fundamentally, however, the opacity in the training process and the very nature of public datasets, particularly within the clinical domain where the availability of such datasets is limited, inherently suggests that any publicly available clinical benchmark dataset is likely to have been seen as part of the model training process. One approach to solving this problem, beyond introducing our permutations, is localizing the MCQA problems into real-world clinical cases from local EHRs with local data representations. Such an approach would serve the dual purpose of both making the assessment more reflective of local performance, and sufficiently permute the syntax of the question to prevent memorization-based solves.

Collectively, these adaptations would likely help mitigate the memorization issue and serve as a relatively straightforward adaptation towards testing application of retained knowledge towards problem solving, rather than rote repetition. We leave such investigations to future work.

### Limitations

Human consistency is not perfect. For example, we would expect that were we to add a “none of the above” option in a manner similar to Setting 3 without removing the correct answer, humans would have substantial inconsistency due to human learned MCQA testing strategies. While infeasible to completely control, we note that our adversarial perturbations challenge human strengths and model weaknesses. The “bottleneck” for humans is knowledge retention, which is the strength of data-driven algorithms. Conversely, while data-driven algorithms encode vast volumes of knowledge, said knowledge is not consistently applied in problem solving, which is generally an issue of much lesser magnitude for humans. We generally expect humans to perform well on our tested permutations, particularly where original context space is provided.

To control for question-difficulty effects, our 100-question subsets were sampled from questions correctly answered by an intersection of models. This sampling approach biases toward “easier” questions, leading to inflated consistency (empirically confirmed during post-hoc merging of new models into the sample set). To ablate the influence of instruction format and obviate need for expensive domain expertise, we have preserved a multiple-choice format throughout these experiments, instead of allowing for free-form generation. Fundamentally, due to these factors, the real-world consistency with MCQA-based results may be worse our results suggest, further highlighting the need for additional adaptation to be more reflective of real-world performance.

## Conclusion

In this study, we aimed to evaluate to what extent correct answers in a traditional MCQA setting generalize when assumptions underpinning MCQA generalizability are invalidated. In this regard, we identified substantial concerns about MCQA response consistency, particularly in smaller models. Our observed inconsistencies significantly challenge the validity of clinical MCQAs as reliable assessment tools for generative language models and show that reasoning can actually harm, rather than help, when these underlying consistency issues are not addressed. These observations underline the necessity of refining existing MCQA benchmarks to better reflect real-world generalizability requirements, and we suggest several approaches by which to do so.

## Methods

### Baseline Assessments, Models and Datasets

We establish the baseline accuracy of eight different models, five with “reasoning” capabilities (defined as the inclusion of reasoning processes during model training^24,25^: GPT-o1^25^, GPT-o1-mini^25^, DeepSeek-R1-Distill-Llama-70B^24^, DeepSeek-R1-Distill-Llama-8B^24^, QwQ-32B^26^) and three without (LLaMA-3.3-70B-Instruct^27^, LLaMA-3.1-8B-Instruct^27^, Qwen-2.5-32B-Instruct^28^), on four different multiple-choice clinical assessment benchmarks. These three non-reasoning models were specifically selected as they are the base models from which reasoning versions are tuned (DeepSeek-R1-Distill-Llama-70B, DeepSeek-R1-Distill-Llama-8B, and QwQ-32B respectively), ablating the reasoning process. Further, we select the R1/LLaMA-8B and GPT-o1-mini models to ablate the influence of parameter sizes in both reasoning and non-reasoning models. The datasets assessed were the test subsets of the MMLU professional medicine dataset^19,29^, MedQA (USMLE Step 2&3 subset)^18^, MedBullets^30^, and the JAMA clinical challenge dataset^30,31^.

A temperature of 0.6 (recommended default) was used for all models supporting the parameter. Each model’s “final answer” to a question is determined by a majority vote across five independently generated outputs. Responses that extended beyond one character were manually normalized (we opted to not use constrained decoding^32^ to avoid confounding influences on output generation). Situations where the output returns multiple answers e.g., “A, B, or D,” are marked incorrect. In cases where generation results in a tie the response is counted as a multiple answer response. GPT-based models were served via Azure OpenAI API version 2024-12-01-preview. All other models were served through vLLM^33^ in native precision using eight NVIDIA H100 80GB GPUs.

To testing consistency amid assumption invalidation, a set of questions that models were able to answer correctly was sampled. To control for varying question difficulty, for the five large (> = 32B parameters) models, we sample 100 questions from the intersection set of correctly answered questions from these five models for subsequent experiments. For the small (8B parameter and GPT-o1-mini) models, we instead use the sample from the intersection set of all eight models, excluding these three from the large model set due to the small number of correct answers reducing the statistical robustness of the sample.

### Assessing Reasoning Fidelity and Semantic Consistency

We categorize our experiments into three experimental settings, observing answer consistency when each of the tested baseline assumptions are invalidated. Each setting is further divided into two sub-experiments, one retaining and one obfuscating or occluding contextual cues. Please refer to Fig. 1 for prompts used.

#### Setting 1: Data Leakage/Memorization amid Expanded Context Spaces:

Answer memorization is not a concern for human examinees due to question volume but is a concern for data-driven algorithms. Opacity on the data used for model training purposes raises significant concerns on answer memorization^34,35^. To assess whether this is occurring, we shuffle the options^36^. **To obfuscate the context**, we expand the search space from the 4–5 options present in the benchmark to an expanded search space of exactly 26 options (A-Z, to ablate influence of multiple-choice option binding). Additional options are sampled from the MedQA train set with subsequent random shuffling (to move correct answers out of A-E). Manual review of mismatches was done for answers not in original option set.

#### Setting 2: Answer Consistency Despite Syntactic Perturbations amid Context Clue Removal:

MCQA assessments assume that an examinee’s answers will be consistent given the same semantic information even if the syntax by which the question is posed changes. In this setting, we aim to assess the validity of this assumption by providing the answer as part of the prompt itself (alongside other possible options in the option list), permuting the question into a true/false assessment. **To occlude this context**, we truncate the other possible options from the prompt.

#### Setting 3: Statistical Predisposition to Positive Answer Matching amid Prompt-Implied Context Loss:

MCQAs assume that knowledge is globally applicable to reasoning processes. Unlike human examinees, however, models rely on statistical predisposition to select answers by matching highly probable terms within provided options. To assess whether this assumption generalizes, we remove the correct answer from the listed multiple-choice options and replace it with “E. None of the above.”. For context obfuscation, the prompt being in the form of a multiple-choice question inherently predisposes models to look for the answer within the context of the A-E option list. **To hide the correct answer from this implied context**, we remove the option “none of the above” and instead insert an instruction to respond as such if none of the listed options listed A-C/D are correct.

## Supplementary Material

This is a list of supplementary files associated with this preprint. Click to download.

- Appendix.docx

## Figures and Tables

**Table 1:** Model Baseline Accuracy on Experimental Datasets

	Large Model					Small Model		
Dataset	GPT-o1	R1-LLaMA-70B	LLaMA-70B	QwQ-32B	Qwen-32B	GPT-o1-Mini	R1-LLaMA-8B	LLaMA-8B
MMLU	264/271 (97.42%)	260/271 (95.94%)	247/271 (91.14%)	254/271 (93.73%)	240/271 (88.56%)	255/271 (94.10%)	198/271 (73.06%)	207/271 (76.38%)
MedQA	545/594 (91.75%)	496/594 (83.50%)	427/594 (71.89%)	475/594 (79.97%)	383/594 (64.48%)	496/594 (83.50%)	322/594 (54.21%)	328/594 (55.22%)
MedBullets	257/298 (86.24%)	217/298 (72.82%)	180/298 (60.4%)	202/298 (67.79%)	144/298 (48.32%)	217/298 (72.82%)	122/298 (40.94%)	128/298 (42.95%)
JAMA	1202/1511 (79.55%)	994/1511 (65.78%)	983/1511 (65.06%)	971/1511 (64.26%)	895/1511 (59.23%)	994/1511 (65.78%)	735/1511 (48.64%)	721/1511 (47.72%)

Generally, large models tend to perform well on Setting 1, although the expansion to 26 options does cause some consistency loss for non-reasoning models. The drop in consistency is however much more pronounced for small models (denoted with a *), in both the original and expanded search space.

## Data Availability

All benchmarks used in this study are sourced from publicly available benchmarks. Due to individual license restrictions, we do not distribute these benchmarks with the code but have left instructions on how to obtain/format said datasets within our GitHub repository’s README

## Code Availability Statement

Code used for the experiments presented in this study can be found on GitHub, at https://github.com/OHNLP/ContextVsReasoningMCQA Note that it is expected that users possess their own OpenAI and HuggingFace tokens authorizing access to the respective models used in this study.

## References

[1] Thirunavukarasu AJ, Ting DSJ, Elangovan K, Gutierrez L, Tan TF, Ting DSW. Large language models in medicine. Nat Med 2023;29(8):1930–1940. (In eng). DOI: 10.1038/s41591-023-02448-8. 37460753

[2] Bedi S, Liu Y, Orr-Ewing L, Testing and Evaluation of Health Care Applications of Large Language Models: A Systematic Review. Jama 2025;333(4):319–328. (In eng). DOI: 10.1001/jama.2024.21700. 39405325 PMC11480901

[3] Yuan J, Zhang J, Wen A, Hu X. The Science of Evaluating Foundation Models. arXiv preprint arXiv:250209670 2025.

[4] Yosinski J, Clune J, Bengio Y, Lipson H. How transferable are features in deep neural networks? Advances in neural information processing systems 2014;27.

[5] Wang A, Singh A, Michael J, Hill F, Levy O, Bowman SR. GLUE: A multi-task benchmark and analysis platform for natural language understanding. arXiv preprint arXiv:180407461 2018.

[6] Devlin J, Chang M-W, Lee K, Toutanova K. Bert: Pre-training of deep bidirectional transformers for language understanding. Proceedings of the 2019 conference of the North American chapter of the association for computational linguistics: human language technologies, volume 1 (long and short papers) 2019:4171–4186.

[7] Wei Q, Chen Y, Salimi M, Cost-aware active learning for named entity recognition in clinical text. J Am Med Inform Assoc 2019;26(11):1314–1322. DOI: 10.1093/jamia/ocz102. 31294792 PMC6798575

[8] Liu J, Wong ZSY. Utilizing active learning strategies in machine-assisted annotation for clinical named entity recognition: a comprehensive analysis considering annotation costs and target effectiveness. J Am Med Inform Assoc 2024;31(11):2632–2640. DOI: 10.1093/jamia/ocae197. 39081233 PMC11491619

[9] Schick T, Schütze H. It’s not just size that matters: Small language models are also few-shot learners. arXiv preprint arXiv:200907118 2020.

[10] Wang F, Zhang Z, Zhang X, A comprehensive survey of small language models in the era of large language models: Techniques, enhancements, applications, collaboration with llms, and trustworthiness. arXiv preprint arXiv:241103350 2024.

[11] Kim H, Hwang H, Lee J, Small language models learn enhanced reasoning skills from medical textbooks. NPJ Digit Med 2025;8(1):240. DOI: 10.1038/s41746-025-01653-8. 40316765 PMC12048634

[12] Qiu J, Lam K, Li G, LLM-based agentic systems in medicine and healthcare. Nature Machine Intelligence 2024;6(12):1418–1420. DOI: 10.1038/s42256-024-00944-1.

[13] Chang C-Y, Jiang Z, Rakesh V, MAIN-RAG: Multi-Agent Filtering Retrieval-Augmented Generation. arXiv preprint arXiv:250100332 2024.

[14] Zou J, Topol EJ. The rise of agentic AI teammates in medicine. The Lancet 2025;405(10477):457. 10.1016/S0140-6736(25)00202-8 39922663

[15] Suura SR. Agentic AI Systems in Organ Health Management: Early Detection of Rejection in Transplant Patients. Journal of Neonatal Surgery 2025;14(4s).

[16] Yang J, Jin H, Tang R, Harnessing the power of llms in practice: A survey on chatgpt and beyond. ACM Transactions on Knowledge Discovery from Data 2024;18(6):1–32.

[17] Haist SA, Katsufrakis PJ, Dillon GF. The Evolution of the United States Medical Licensing Examination (USMLE): Enhancing Assessment of Practice-Related Competencies. JAMA 2013;310(21):2245–2246. DOI: 10.1001/jama.2013.282328. 24302081

[18] Jin D, Pan E, Oufattole N, Weng W-H, Fang H, Szolovits P. What disease does this patient have? a large-scale open domain question answering dataset from medical exams. Applied Sciences 2021;11(14):6421.

[19] Hendrycks D, Burns C, Basart S, Measuring massive multitask language understanding. arXiv preprint arXiv:200903300 2020.

[20] Raji ID, Daneshjou R, Alsentzer E. It’s Time to Bench the Medical Exam Benchmark. Nejm Ai 2025;2(2). DOI: 10.1056/AIe2401235.

[21] Li W, Li L, Xiang T, Liu X, Deng W, Garcia N. Can multiple-choice questions really be useful in detecting the abilities of LLMs? arXiv preprint arXiv:240317752 2024.

[22] Matton K, Ness R, Guttag J, Kiciman E. Walk the Talk? Measuring the Faithfulness of Large Language Model Explanations. The Thirteenth International Conference on Learning Representations.

[23] Lanham T, Chen A, Radhakrishnan A, Measuring faithfulness in chain-of-thought reasoning. arXiv preprint arXiv:230713702 2023.

[24] Guo D, Yang D, Zhang H, Deepseek-r1: Incentivizing reasoning capability in llms via reinforcement learning. arXiv preprint arXiv:250112948 2025.

[25] Jaech A, Kalai A, Lerer A, Openai o1 system card. arXiv preprint arXiv:241216720 2024.

[26] Team Qwen. Qwq-32b: Embracing the power of reinforcement learning. URL: https://qwenlmgithubio/blog/qwq-32b 2025.

[27] Grattafiori A, Dubey A, Jauhri A, The llama 3 herd of models. arXiv preprint arXiv:240721783 2024.

[28] Yang A, Yang B, Zhang B, Qwen2. 5 technical report. arXiv preprint arXiv:241215115 2024.

[29] Gema AP, Leang JOJ, Hong G, Are We Done with MMLU? arXiv preprint arXiv:240604127 2024.

[30] Chen H, Fang Z, Singla Y, Dredze M. Benchmarking large language models on answering and explaining challenging medical questions. arXiv preprint arXiv:240218060 2024.

[31] Chang HJ, Fontanarosa PB. Introducing the JAMA Clinical Challenge. JAMA 2011;305(18):1910–1910. DOI: 10.1001/jama.2011.625.

[32] Beurer-Kellner L, Fischer M, Vechev M. Guiding llms the right way: Fast, non-invasive constrained generation. arXiv preprint arXiv:240306988 2024.

[33] Kwon W, Li Z, Zhuang S, Efficient memory management for large language model serving with pagedattention. Proceedings of the 29th Symposium on Operating Systems Principles 2023:611–626.

[34] Dong Y, Jiang X, Liu H, Generalization or memorization: Data contamination and trustworthy evaluation for large language models. arXiv preprint arXiv:240215938 2024.

[35] Sainz O, Campos JA, García-Ferrero I, Etxaniz J, de Lacalle OL, Agirre E. NLP evaluation in trouble: On the need to measure LLM data contamination for each benchmark. arXiv preprint arXiv:231018018 2023.

[36] Zong Y, Yu T, Chavhan R, Zhao B, Hospedales T. Fool your (vision and) language model with embarrassingly simple permutations. arXiv preprint arXiv:231001651 2023.

[37] Gilson A, Safranek CW, Huang T, How Does ChatGPT Perform on the United States Medical Licensing Examination (USMLE)? The Implications of Large Language Models for Medical Education and Knowledge Assessment. JMIR Med Educ 2023;9:e45312. DOI: 10.2196/45312. 36753318 PMC9947764

[38] Yaneva V, Baldwin P, Jurich DP, Swygert K, Clauser BE. Examining ChatGPT Performance on USMLE Sample Items and Implications for Assessment. Acad Med 2024;99(2):192–197. DOI: 10.1097/ACM.0000000000005549. 37934828 PMC11444356

